# The CaMKII K42M and K42R mutations are equivalent in suppressing kinase activity and targeting

**DOI:** 10.1371/journal.pone.0236478

**Published:** 2020-07-27

**Authors:** Jonathan E. Tullis, Nicole L. Rumian, Carolyn Nicole Brown, K. Ulrich Bayer

**Affiliations:** 1 Department of Pharmacology, University of Colorado Anschutz Medical Campus, Aurora, CO, United States of America; 2 Program in Neuroscience, University of Colorado Anschutz Medical Campus, Aurora, CO, United States of America; University of South Alabama, UNITED STATES

## Abstract

CaMKII is an important mediator of forms of synaptic plasticity that are thought to underly learning and memory. The CaMKII mutants K42M and K42R have been used interchangeably as research tools, although some reported phenotypic differences suggest that they may differ in the extent to which they impair ATP binding. Here, we directly compared the two mutations at the high ATP concentrations that exist within cells (~4 mM). We found that both mutations equally blocked GluA1 phosphorylation *in vitro* and GluN2B binding within cells. Both mutations also reduced but did not completely abolish CaMKII T286 autophosphorylation *in vitro* or CaMKII movement to excitatory synapses in neurons. Thus, despite previously suggested differences, both mutations appear to interfere with ATP binding to the same extent.

## Introduction

**Fig 1:**
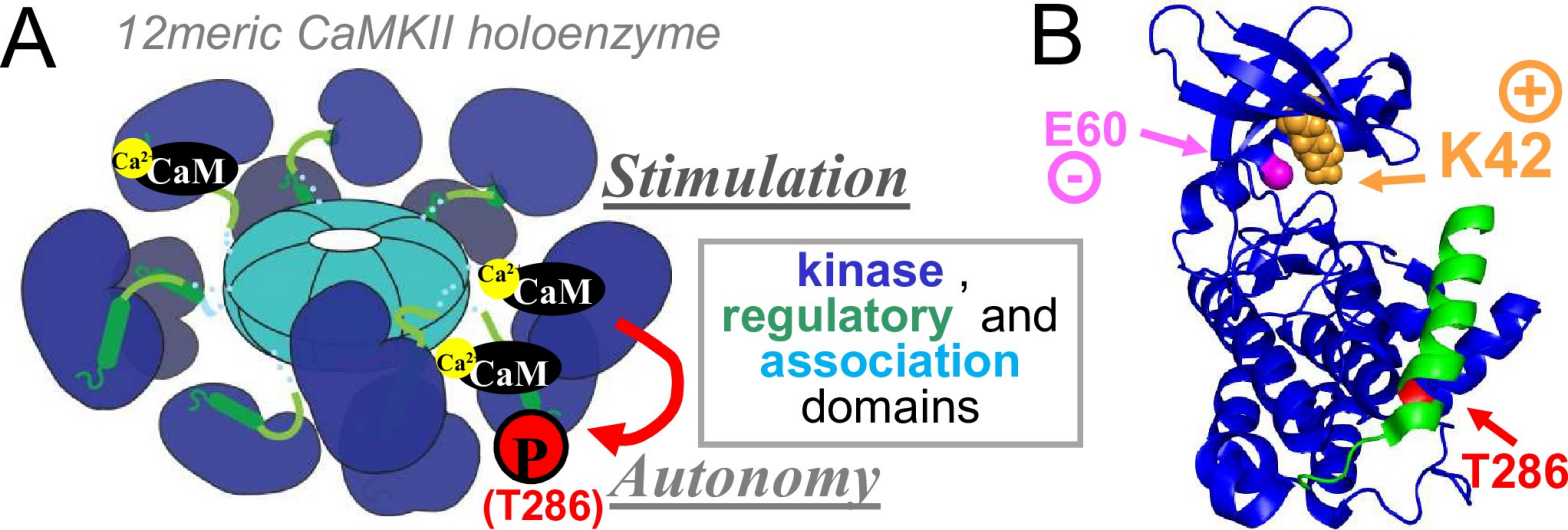
CaMKII structure and regulation. (A) CaMKII forms 12meric holoenzymes, with each subunit containing an N-terminal kinase domain (blue), regulatory domain (green) and C-terminal association domain (aqua). Direct Ca^2+^/CaM binding to each regulatory domain induces stimulated activity of each subunit. A Ca^2+^/CaM-dependent inter-subunit autophosphorylation at T286 generated Ca^2+^-independent autonomous activity. Either Ca^2+^/CaM or T286 phosphorylation is sufficient to induce binding to GluN2B, at least when nucleotide is present. (B) Ribbon structure of the kinase (blue) and regulatory (green) domain. K42 (orange) on β-sheet 3 helps form the nucleotide binding pocket by interacting with residue E60 (magenta) on α-helix C and with the α- and β-phosphates of ATP or ADP (not shown).

The Ca^2+^/calmodulin(CaM)-dependent protein kinase II (CaMKII; Fig 1) is a major mediator of higher brain functions such as learning and memory, as well as of the underlying forms of synaptic plasticity, specifically including long-term potentiation (LTP) of excitatory glutamatergic synapses [1–4]. Normal LTP is expressed largely by potentiation of synaptic AMPA-type glutamate receptors (AMPARs) [5–7] and is thought to require (i) Ca^2+^-stimulated CaMKII activity [8–10], (ii) the CaMKII T286 autophosphorylation that generates Ca^2+^-independent “autonomous” activity [11, 12], and (iii) the CaMKII binding to the NMDA-type glutamate receptor (NMDAR) subunit GluN2B that underlies much of the CaMKII targeting to excitatory synapses [13–16]. All three of these functions require nucleotide binding to CaMKII: Whereas ATP binding is an obvious requirement for kinase activity (including autophosphorylation), nucleotide binding is additionally required for efficient binding to GluN2B [17–19]. In case of GluN2B binding, ATP can be substituted for by other nucleotides such as ADP or AMP-PNP [17], or even by the nucleotide-competitive inhibitors staurosporine or H7 [18].

In order to genetically abolish CaMKII activity for functional studies, mutations of its lysine residue 42 (K42) have been utilized [20–26]. Homologues of K42, such as K72 in PKA, are found in every active kinase (in β-sheet 3), as it is involved in nucleotide binding by two mechanisms: (i) it directly interacts with ATP or ADP (by interacting with the negatively charged α- and β-phosphates) and (ii) it helps shape the overall nucleotide-binding pocket by interactions with a negatively charged glutamic acid residue (on α-helix C; see Fig 1B). Mutations of CaMKII K42 to methionine (K42M) or arginine (K42R) have been used interchangeably. However, K and R share the positive charge that mediates the crucial interactions for the nucleotide binding. Thus, even though the K42R should be expected to significantly reduce ATP binding, it might not completely eliminate it. Notably, due to the high concentration of ATP within cells (~4 mM), even a 100-fold decrease in the CaMKII affinity for ATP (i.e. K_M_ increase from ~8 μM to 0.8 mM) would not be sufficient to dramatically reduce kinase activity in cellular conditions. Furthermore, whereas the K42M mutant has been described to disrupt CaMKII binding to GluN2B and localization to excitatory synapses [17], the K42R mutant has been described to allow the GluN2B-mediated CaMKII movement to excitatory synapses [23].

Here, we directly compared the K42R and K42M mutants for effects *in vitro*, in heterologous cells, and in hippocampal neurons. Our results indicate equal inhibition of ATP- effects for both mutants: Both mutations blocked S831 phosphorylation of the AMPAR subunit GluA1 *in vitro* and the Ca^2+^-induced binding to GluN2B in HEK cells. Both mutations also much reduced but did not completely eliminate CaMKII T286 autophosphorylation *in vitro* or glutamate-induced movement to synapses in neurons.

## Materials and methods

### Ethical statement

No live animal experiments were performed. For hippocampal cultures, P0-P1 neonatal rat pups of both sexes were used. Pregnant Sprague-Dawley rats were supplied by Charles River Labs. All animal treatment for this study was approved by the Institutional Animal Care and Use Committee of the University of Colorado Anschutz Medical Campus.

### Constructs and protein preparations

Mammalian expression vectors for GFP-CaMKIIα [22, 27], shRNA for CaMKII knockdown [28], pDisplay-mCherry-GluN2Bc (containing the GluN2B cytoplasmic C-tail from amino acids 1122 to 1482) [29], labelled intrabody for PSD95 [30, 31], and bacterial expression vector for GST-GluA1 C-tail [32] were described previously.

GFP-CaMKIIα WT and the K42 mutants were harvested from HEK-293 cells as previously described [33]. For comparison, CaMKII concentrations were evaluated via Western Blot. Protein extracts were then supplemented with untransfected HEK-293 cell extract to normalize content of total protein. GST-GluA1 C-tail was purified after bacterial expression as previously described [32].

### Western blot analysis of CaMKII activity assay

CaMKII activity was measured by *in vitro* phosphorylation of purified GST-GluA1 at Ser-831. Reactions contained 10 nM CaMKII (subunit concentration), 1 μM GST-GluA1, 50 mM PIPES pH 7.1, 2 mM CaCl_2_, 10 mM MgCl_2_, 1 μM calmodulin, 4 mM ATP, and 2 μM of the phosphatase inhibitor microcystin. Reactions were done at 30°C for 1 or 5 min, and stopped by adding SDS-loading buffer containing 1 mM EDTA followed by incubation in a boiling water bath for 10 min. Samples were then analyzed via Western Blot for GST, phospho-S831, CaMKII, and phospho-T286, essentially as we have described previously [32, 34].

### GluN2B colocalization in HEK cells

HEK-293 cells were transfected by the calcium phosphate method with pDisplay-mCh-GluN2Bc and GFP-CaMKII WT, K42M, or K42R for 16–24 hours. Images from three independent cultures were collected at 32°C in HEPES buffered imaging solution as in the neuronal imaging experiments using 0.5 μm steps over 4 μm of the cell center. Cells were imaged before and 10 min after stimulation with 10 μM ionomycin. 2D maximum intensity projection images were generated and analyzed using ImageJ software. A threshold of the mCh signal above background was acquired, and a Pearson’s correlation of fluorescent overlap for each time point was calculated. Raw Pearson’s correlations are shown.

### Synaptic localization in dissociated hippocampal cultures

Image acquisition and analysis: DIV 15–18 rat neuronal cultures were transfected for 24–48 hours with shRNA for CaMKII 5’UTR to knock down endogenous CaMKII, mCh-PSD95 intrabody, GFP-CaMKII, and an iRFP empty vector as a cell fill. Images were collected at 32°C in HEPES buffered imaging solution containing (in mM) 130 NaCl, 5 KCl, 10 HEPES pH 7.4, 20 Glucose, 2 CaCl_2_, 1 MgCl_2_, adjusted to proper osmolarity with sucrose. Images of individual neurons from two independent cultures were acquired by 0.5 μm steps over 6 μm. 2D maximum intensity projection images were then generated and analyzed using a custom-build program in ImageJ. The program utilizes combinatorial thresholding to mask regions of the cell that contain high intensity PSD-95 puncta (the post-synaptic side of excitatory synapses in dendritic spines) and regions of the dendritic shaft that contain no fluorescence intensity of PSD-95. The program then takes the ratio of average CaMKII fluorescence intensity of the PSD-95 mask to the average CaMKII fluorescence intensity in the dendritic shaft mask as a measure of synaptic enrichment. Note that using the average intensities makes the ratio independent of the mask areas.

Neuronal cultures were stimulated by bath application of 100 μM glutamate and 10 μM glycine for 1 min, which induces robust CaMKII accumulation at excitatory synapses [13, 17, 22, 27, 31, 35] and has been previously shown to increase AMPAR surface expression [35]. This stimulus was applied after the first image, and then washed out with 5 volumes of fresh imaging solution to allow for post-stimulus timepoints of 1 and 5 min to be examined.

## Results

### The CaMKII K42M and K42R mutations prevent GluA1 phosphorylation *in vitro*

**Fig 2:**
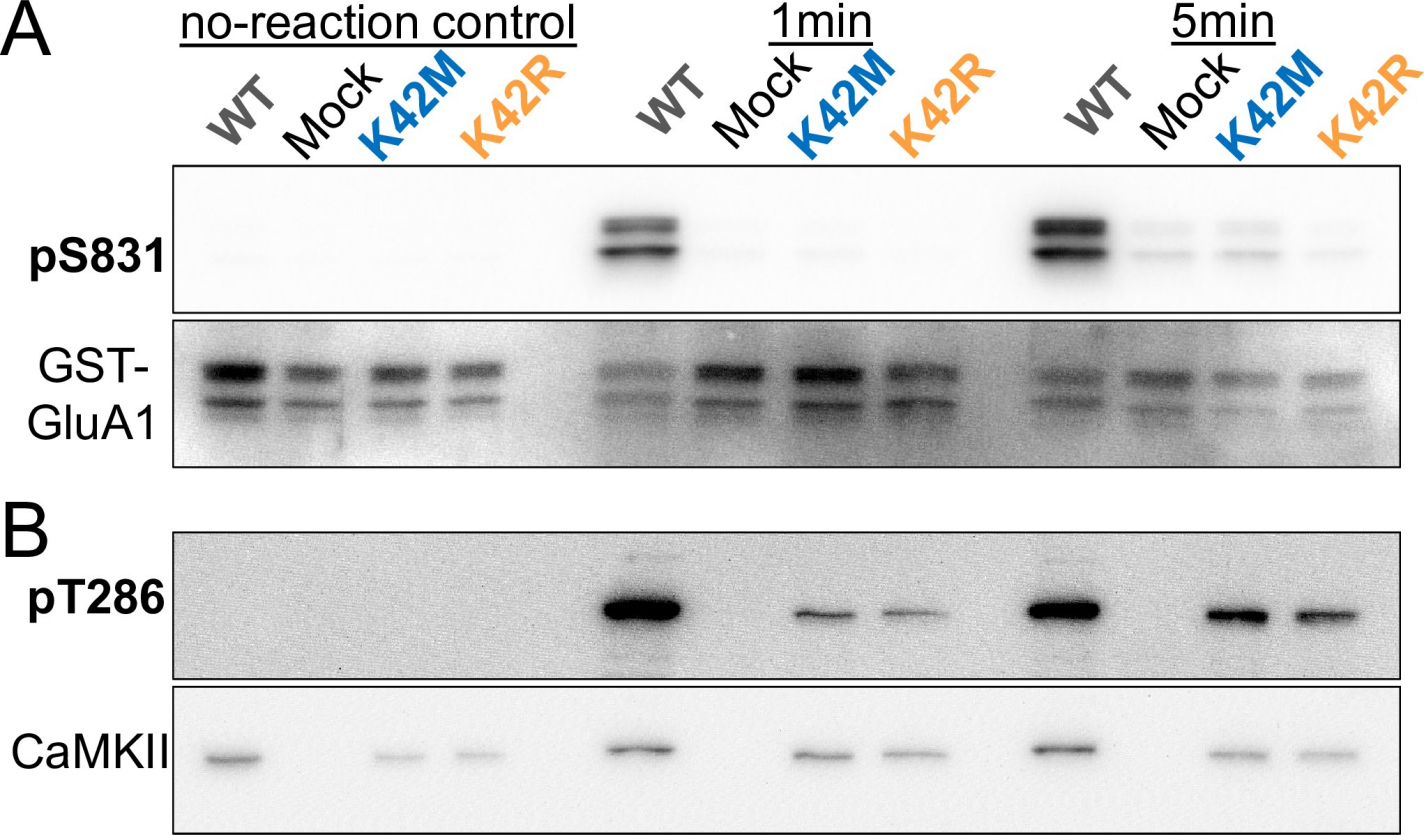
The K42M and K42R mutations impair CaMKII activity *in vitro* even at high ATP concentrations. (4 mM). Reactions were carried out for 1 min or 5 min at 30°C (or no-reaction control for 0 min on ice), and phosphorylation was detected by Western blot. (A) Both K42 mutations cause almost complete block of the phosphorylation of an exogenous substrate, S831 of the GluA1 cytoplasmic C-tail (purfied as GST-fusion protein after expression in bacteria). (B) Both K42 mutations reduce but do not completely block the fast CaMKII autophosphorylation at T286 in the same reactions.

Both K42M and K42R mutants were expected to reduce ATP binding by significantly increasing the K_M_ for ATP (~8 μM for CaMKII wild type). Here, we decided to determine if reduction in ATP binding is sufficient to block kinase activity for both mutants also at high ATP concentrations (4 mM, i.e. the approximate typical concentration found within cells). We performed *in vitro* reactions with CaMKII wild type versus K42M mutants that were expressed in HEK cells, and with a GST-fusion protein of the GluA1 C-tail as substrate, followed by Western blot analysis of phosphorylation of GluA1 at S831 (Fig 2A and S1 Fig), a CaMKII-site know to mediate LTP-related increase in single channels conductance [36–38]. In control conditions without kinase reaction (i.e. no ATP and no incubation at 30°C), the antibody did not detect any S831 phosphorylation. After 1 min of kinase reaction time (at 30°C), a strong S831 phosphorylation signal was detected only for CaMKII wild type (Fig 2A). Even after a prolonged 5 min kinase reaction time, the K42M and K42R mutants still yielded only very faint signals, and a similar faint signal was also observed in the no-kinase control with mock-transfected HEK cell extract (Fig 2A). These results indicate that the K42M and K42R mutant block CaMKII-mediated substrate phosphorylation equally and effectively.

### Residual T286 phosphorylation in the CaMKII K42M and K42R mutants

Next, we compared the two K42 mutants for CaMKII autophosphorylation at T286. For this purpose, we re-probed the Western blots from the phospho-S831 analysis with a corresponding anti-phospho-T286 antibody (Fig 2B and S1 Fig). In contrast to S831 phosphorylation, T286 autophosphorylation by CaMKII wild type was complete already at 1 min and did not further increase by prolonged 5 min reaction time (Fig 2B), consistent with the fast speed of this autophosphorylation that rapidly depletes substrates to phosphorylate within the CaMKII holoenzyme [39]. In contrast to S831 phosphorylation, substantial T286 autophosphorylation was seen also for both of the K42 mutants, although to a substantially lesser degree the for CaMKII wild type (Fig 2B). This indicates that there could be some level of residual ATP binding in both of the K42 mutants. However, most importantly, the K42R and K42M mutants do not appear to differ in the level of any potential residual ATP binding.

### CaMKII K42M and K42R mutations block the Ca^2+^-induced binding to GluN2B

**Fig 3:**
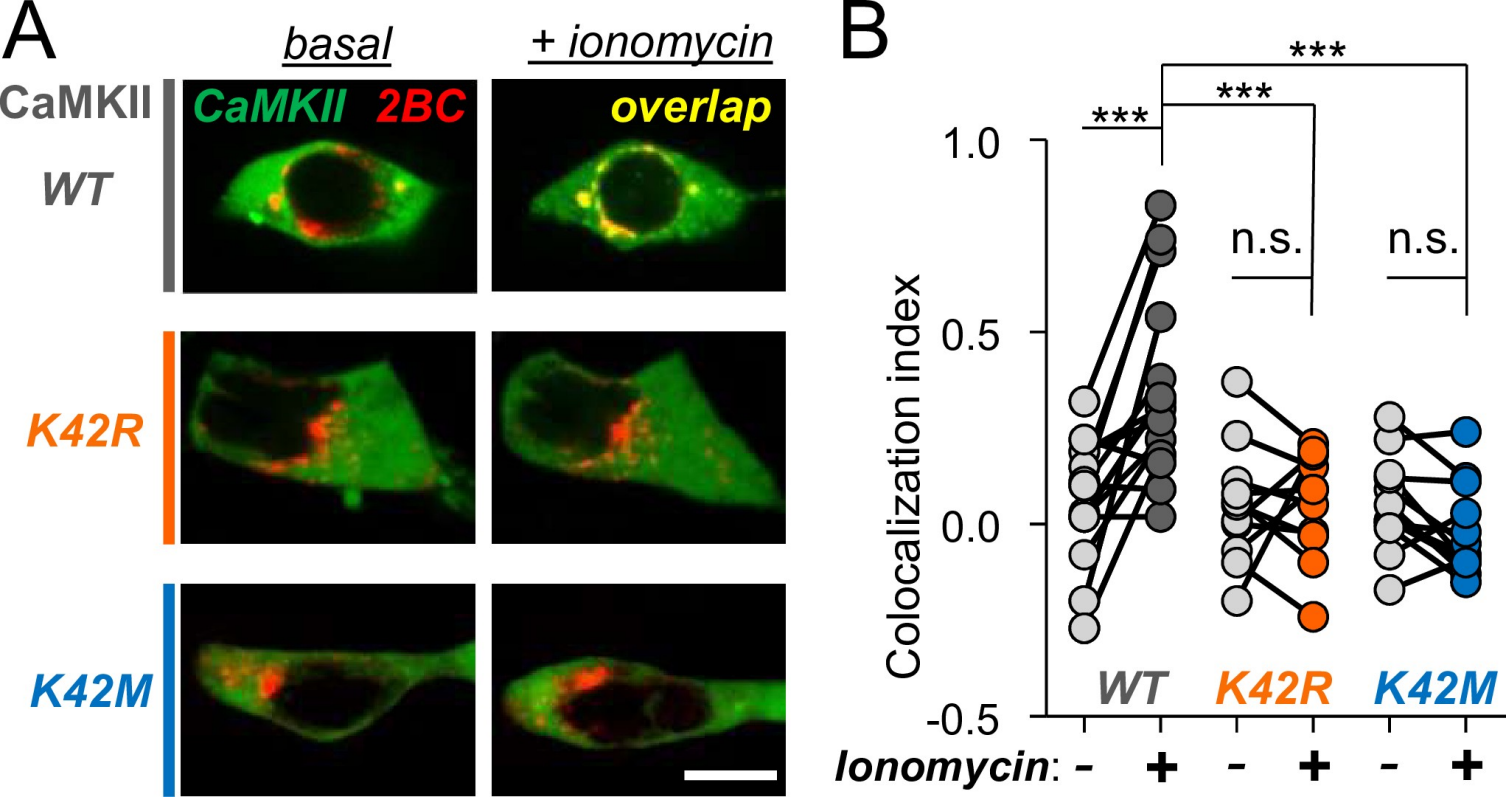
The K42M and K42R mutations prevent CaMKII binding to GluN2B in a cellular co-localization assay. GFP-CaMKII was co-expressed with mCherry-labeled membrane-targeted GluN2Bc in HEK-293 cells. Co-localization was stimulated by inducing Ca^2+^ signals with 10 μM ionomycin, but only for CaMKII wild type (WT; n = 23) and not for either the K42R (n = 11) or K42M (n = 12) mutants. (A) Example images before and 10 min after a 1 min ionomycin treatment. Scale bar: 10 μm. (B) Quantification of the Pearsons correlation of co-localization, shown paired before and after stimulation for each cell. ***: p<0.001; n.s.: not significant, two-way ANOVA with Bonferroni post-hoc analysis.

Nucleotide binding to CaMKII is required not only for kinase activity, but also for efficient Ca^2+^/CaM-induced binding to the NMDAR subunit GluN2B (in a manner that is independent from kinase activity) [17, 18]. Thus, we compared CaMKII wild type and the two K42 mutants in our established GluN2B co-localization assay after expression in HEK cells. For this assay, GFP-CaMKII is co-expressed together with an mCherry fusion protein containing a membrane anchor and the cytoplasmic GluN2B C-tail; co-localization is induced by triggering a Ca^2+^-stimulus with ionomycin [29, 40]. Without stimulation, little or no co-localization with mCherry-GluN2B was observed for any of the GFP-CaMKII constructs (Fig 3A), as expected. After ionomycin treatment, a significant increase in co-localization was seen, but only for GFP-CaMKII wild type and not at all for either of the two K42 mutants (Fig 3A and 3B). Thus, both the K42M and the K42R mutations completely block the Ca^2+^-induced binding to GluN2B, even at the high ATP concentrations within cells.

### CaMKII K42M and K42R mutations reduced synaptic enrichment in neurons, but did not completely prevent glutamate-induced CaMKII movement

**Fig 4:**
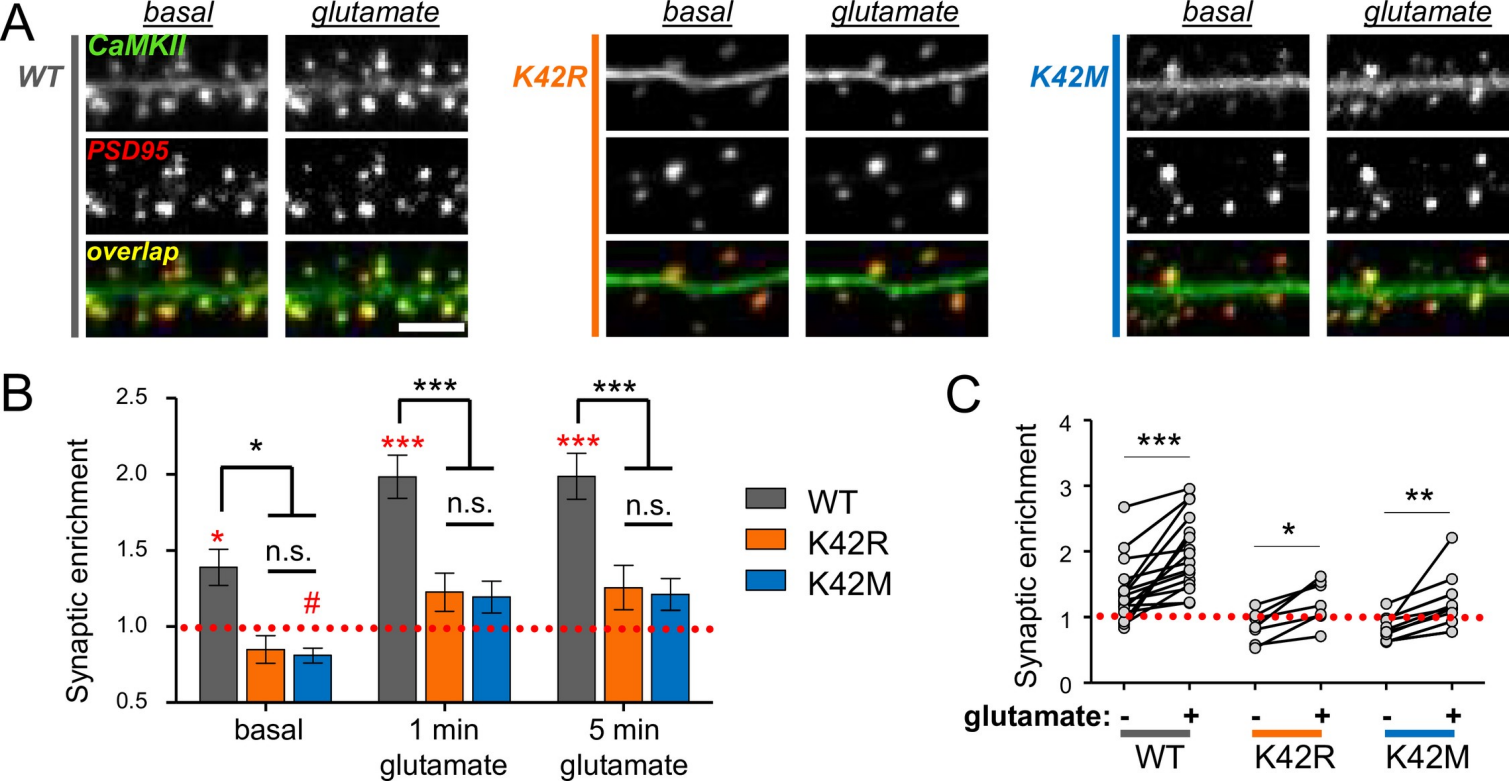
The K42M and K42R mutations reduce synaptic localization of CaMKII in cultured hippocampal neurons. (A) Example images before and after stimulation with 100 μM glutamate (in presence of 10 μM glycine) for 1 min. Scale bar: 4 μm. (B) Column statistics indicate that only CaMKII wild type is significantly synaptically enriched (spine to shaft ratio >1) at any given time point; *: p<0.01; ***: p<0.0001, in one sample t-test (red). The K42M mutant showed even significantly reduced synaptic localization (spine to shaft ratio <1) at one time point; #: p<0.01 (red). At any given time point, CaMKII wild type (n = 16) differed significantly from both the K42R (n = 7) and the K42M (n = 13) mutants, but the K42 mutants did not differ from each other; *: p<0.05; ***: p<0.001; n.s.: not significant, two-way ANOVA with Bonferroni post-hoc analysis (black). (C) Paired illustration of synaptic localization before and after glutamate values in the same neurons, indicating a mild but significant increases in synaptic localization also for the two K42 mutants; *: p<0.05; **: p<0.01; ***: p<0.001 two-way ANOVA with Bonferroni post-hoc analysis.

CaMKII binding to GluN2B is thought to mediate much of the CaMKII targeting to excitatory synapses and its further enrichment in response to LTP stimuli [13–16, 27, 41, 42]. (By contrast, LTD stimuli instead cause CaMKII movement to inhibitory synapses and this is not mediated by GluN2B binding [31, 35]). Thus, we decided to compare CaMKII wild type and the two K42 mutants for their basal localization and their LTP-related glutamate-induced accumulation at excitatory synapses. Excitatory synapses were labeled in live hippocampal neurons by co-expression of fluorescently-tagged intrabodies against the marker protein PSD-95 [30], as we have recently described [31, 43]. As expected, the excitatory synapses were mainly localized to small protrusions from the dendrites that are called dendritic spines (Fig 4A). Endogenous CaMKII was knocked down with our established shRNA [28] and replaced by expressing either GFP-CaMKII wild type or a K42 mutant. (Fig 4A). Before stimulation, GFP-CaMKII wild type localized significantly more to excitatory synapses than any of the K42 mutants (Fig 4A and 4B). Synaptic co-localization with PSD-95 of the two different K42 mutants was indistinguishable from each other (Fig 4A and 4B) and from iRFP that was co-expressed as cell fill (S2 Fig). Thus, the K42R and K42M mutations caused an equally reduced basal localization to excitatory synapses.

After stimulation with glutamate (100 μM for 1 min, in presence of 10 μM of the NMDAR co-agonist glycine), the difference in localization between CaMKII wild type and the K42 mutants was maintained (Fig 4A and 4B). In fact, under any conditions, only CaMKII wild type showed any significant synaptic enrichment (with a spine to shaft ratio >1; Fig 4B). However, as the basal localization of the K42 mutants even showed a slight depletion at excitatory synapses (with a spine to shaft ratio below 1; Fig 4B), glutamate stimuli still induced a small but significant increase in synaptic localization (Fig 4C). No increase in PSD-95 co-localization was observed for the iRFP cell fill (S2 Fig), indicating that the increased synaptic localization of the K42 mutants is not due to changes in dendritic spine morphology. Importantly, this smaller synaptic increase of the mutants was indistinguishable between the K42R and K42M mutants, indicating that it is not caused by any difference between the two distinct K42 mutations.

## Discussion

The results of this study show that the CaMKII K42R and K42M mutations are functionally equivalent in disrupting nucleotide effects on CaMKII (such as kinase activity and GluN2B binding), even at cellular ATP concentrations. Thus, the mutants can be considered equivalent in the interpretation and comparison of the functional results obtained with them. This equivalency is despite both theoretical considerations and some apparently contradictory experimental observations: (i) the conserved positive charge in the K42R mutation raised the possibilities of only partial impairment that could be insufficient for complete block at the high ATP concentrations found within cells, and (ii) the K42M mutant was described to impair the glutamate-induced synaptic CaMKII translocation and the underlying binding to GluN2B [17], whereas the K42R mutant was described to maintain the synaptic translocation [23]. Our results clarify that the apparently different observations are actually consistent with each other: Both K42 mutants block Ca^2+^-induced CaMKII binding to GluN2B in heterologous cells, and both mutants reduce but do not completely block glutamate-induced CaMKII movement to excitatory synapses. Thus, our direct comparison of the two mutants revealed that both previous observations are likely true and still consistent with the equivalency of the mutants: Both K42 mutants do allow significant synaptic CaMKII translocation, even though their synaptic localization is much reduced compared to wild type. However, this also raises an important question: If the glutamate-induced CaMKII movement to synapses is mediated by GluN2B binding [13–16, 27, 41, 42] and the K42 mutants completely block this binding in heterologous cells (as shown previously for K42M [17] and here for both mutants), why do the K42 mutants still show some movement to synapses in neurons? Part of the answer may lie in the fact that CaMKII can also interact with numerous other proteins at excitatory synapses [2, 4, 44, 45]. However, this cannot be the full answer, as completely preventing CaMKII binding to GluN2B completely disrupted the synaptic translocation [13, 15, 27, 43]. Thus, another part of the answer may be that the K42 mutants do not completely block GluN2B binding. Indeed, *in vitro*, the K42M mutant retained ~10% of the GluN2B binding seen with CaMKII wild type [17]. This dramatic reduction in binding may be sufficient to completely block Ca^2+^-induced CaMKII movement to GluN2B in HEK cells (where the membrane-targeted GluN2B is more dispersed and not clustered with other CaMKII binding proteins), but still allow some CaMKII movement to synaptic GluN2B in neurons (where GluN2B is more locally clustered and in vicinity of other CaMKII binding proteins). Clusters of CaMKII binding proteins may enable simultaneous interactions of multiple subunits of a single CaMKII holoenzyme, thereby enhancing overall binding via avidity effects.

The equivalence of the K42M and K42R mutations facilitates comparison of past and future results obtained with them. However, other questions for their use as research tools remain. For many cellular functions, these nucleotide binding-incompetent mutants do not only act as “null” mutants but even as “dominant negatives”. However, the action as dominant negative may depend on the specific cellular function, and thus, this designation cannot be made *a priori*. Further, the mechanism by which the mutants act as dominant negatives may also vary by cellular function. For LTP, incorporating K42 mutants into CaMKII holoenzymes could act dominantly negative by reducing the inter-subunit T286 autophosphorylation or by reducing binding to GluN2B. Remarkably, even transient K42M expression has been described to persistently erase memory (both normal spatial memory [21] and mal-adaptive addiction related memory [20]), and it will be interesting to dissect the underlying mechanisms.

## Supporting information

S1 Fig Raw blot images related to Fig 2. Blots were developed using enhanced chemiluminescent (ECL) HRP substrates (Western Lighting Plus ECL, Perkins Elmer) and imaged using the ChemiImager 4400 system (Alpha-Innotech). Densitometry was calculated in FIJI (NIH). (A) Western blots detecting GluA1 S831 phosphorylation and total GluA1. (B) Western blots detecting CaMKII T286 phosphorylation and total CaMKII. (C) Detection of the stained non-luminescent weight marker proteins under illumination with visible light.
(TIF)

S2 Fig Synaptic localization of iRFP cell fill does not change with CaMKII wild type versus mutant expression or glutamate stimuli. Related to Fig 4, as examples and quantification of the cell fill co-expressed in the same neurons is shown. (A) Example images for PSD-95 (detected by intrabody), GFP-CaMKII, and iRFP detected within a dendritic segment of the same cultured hippocampal neuron. (B) Quantification of the synaptic localization of iRFP indicates that the cell fill is not enriched in synapses, neither in neurons expressing CaMKII wild type nor in neurons expressing either of the two K42 mutants. The glutamate stimuli did not change synaptic localization under either condition (n.s.: not significant in 2-way ANOVA with Bonferoni post test).
(TIF)
