# Supplementary figures and images for: The CaMKII K42M and K42R mutations are equivalent in suppressing kinase activity and targeting
Source: PLoS One. 2020 Jul 27;15(7):e0236478. doi: 10.1371/journal.pone.0236478 (PMC7384616; doi:10.1371/journal.pone.0236478)

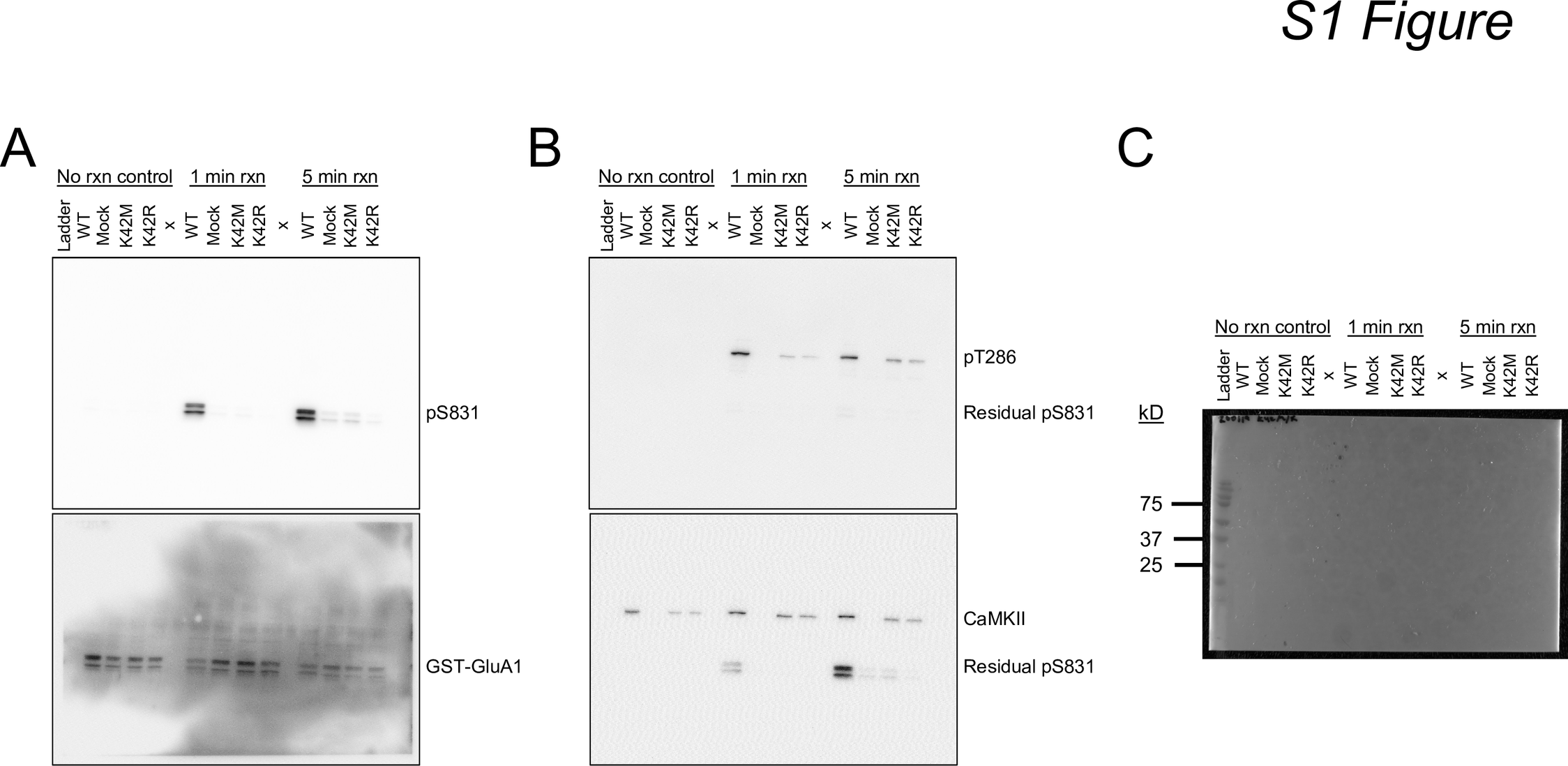

Supplement: S1 Fig — Blots were developed using enhanced chemiluminescent (ECL) HRP substrates (Western Lighting Plus ECL, Perkins Elmer) and imaged using the ChemiImager 4400 system (Alpha-Innotech). Densitometry was calculated in FIJI (NIH). (A) Western blots detecting GluA1 S831 phosphorylation and total GluA1. (B) Western blots detecting CaMKII T286 phosphorylation and total CaMKII. (C) Detection of the stained non-luminescent weight marker proteins under illumination with visible light. (TIF) [file pone.0236478.s001.tif]

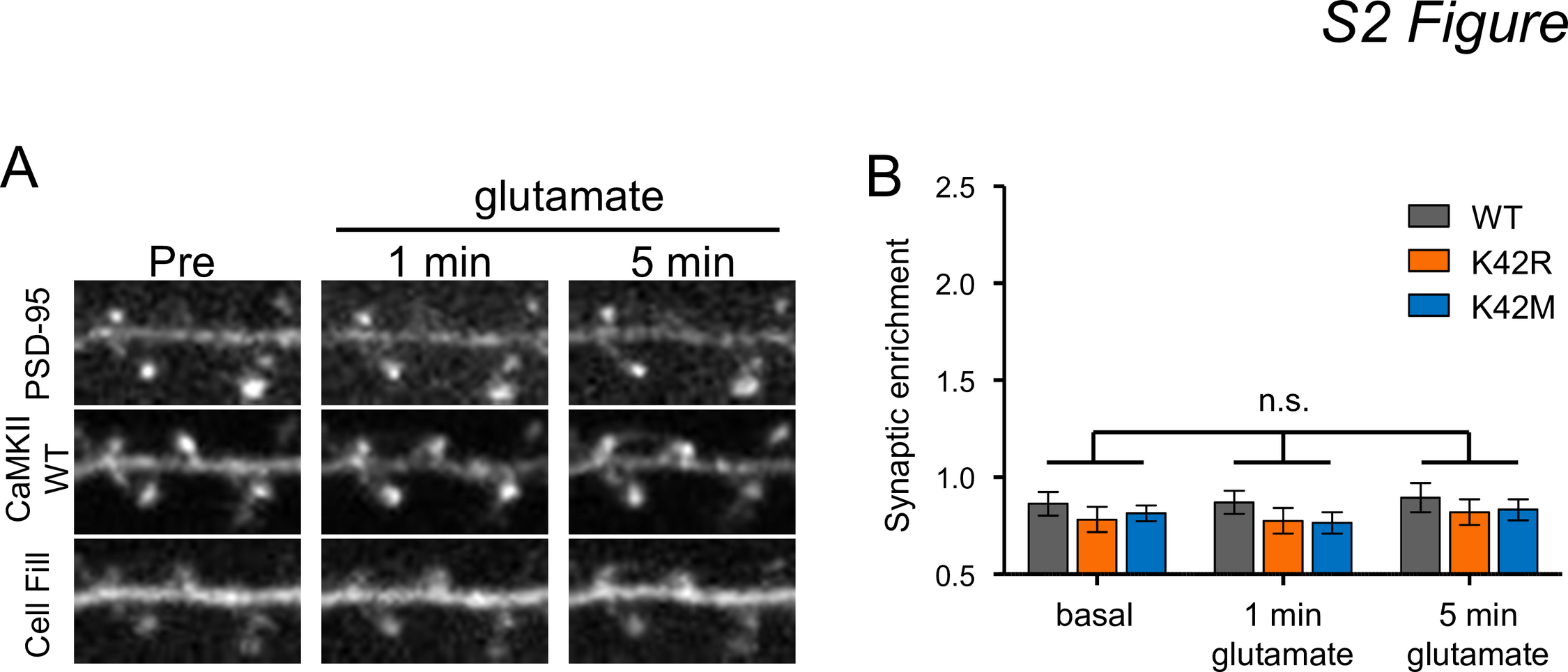

Supplement: S2 Fig — Related to Fig 4, as examples and quantification of the cell fill co-expressed in the same neurons is shown. (A) Example images for PSD-95 (detected by intrabody), GFP-CaMKII, and iRFP detected within a dendritic segment of the same cultured hippocampal neuron. (B) Quantification of the synaptic localization of iRFP indicates that the cell fill is not enriched in synapses, neither in neurons expressing CaMKII wild type nor in neurons expressing either of the two K42 mutants. The glutamate stimuli did not change synaptic localization under either condition (n.s.: not significant in 2-way ANOVA with Bonferoni post test). (TIF) [file pone.0236478.s002.tif]
